# Balancing signals in the intestinal niche

**DOI:** 10.15252/embj.201796431

**Published:** 2017-02-03

**Authors:** Sanne M van Neerven, Louis Vermeulen

**Affiliations:** ^1^ Laboratory of Experimental Oncology and Radiobiology (LEXOR) Center for Experimental Molecular Medicine (CEMM) Academic Medical Center Amsterdam The Netherlands

**Keywords:** Signal Transduction, Stem Cells

## Abstract

During intestinal regeneration, opposing gradients of Wnt and BMP signaling ensure successful differentiation along the crypt/villus axis. In this issue of *The EMBO Journal*, Horiguchi *et al* (2017) show how intestinal subepithelial myofibroblasts can influence cell fate decisions in the regenerating intestine via autocrine secretion of angiopoietin‐like protein 2 (ANGPTL2).

The intestinal tract faces an enormous amount of internal and external stress stimuli on a daily basis. In order to cope with these stress factors and prevent potential damage, the entire epithelium is being replaced every five days. The inner layer of the intestine consists of a single epithelial layer that displays extrusions called “villi” and invaginations known as “crypts”. Regeneration and differentiation of the intestinal epithelium occurs along the crypt/villus axis and is initiated by intestinal stem cells (ISCs) residing at the base of the crypts. Here, the ISCs give rise to transit amplifying precursor cells that can generate a spectrum of functional cell types that move toward the lumen until they are eventually shed. The differentiation status of a cell is determined by its position along the crypt/villus axis, which is tightly regulated by opposing gradients of morphogens such as Wnt and bone morphogenetic protein (BMP). Wnt/β‐catenin signaling is highest at the crypt base, where it promotes stem cell expansion, whereas BMPs are most abundant near the lumen and inhibit proliferation (Vermeulen & Snippert 2014). In addition to these gradients, which are principally maintained by diffusion of ligands, intestinal subepithelial myofibroblasts (ISEMFs) that reside near the crypt base secrete BMP antagonists such as Gremlin1, Gremlin2, and Noggin, thereby promoting stemness at the crypt base (Kosinski *et al*, 2007).

Disruption of these morphogen gradients can have disastrous effects on homeostasis and damage repair and can eventually result in disease. Overexpression of the Wnt pathway by either inactivation of the adenomatous polyposis coli (*APC*) gene or activating mutations in β‐catenin (*CTNNB1*) has been observed in most of the colorectal cancers (CRCs) following the classical adenoma–carcinoma sequence (Vermeulen & Snippert, 2014). In addition, inactivation of BMP receptor 1a (BMPR1A) or Mothers against decapentaplegic homolog 4 (SMAD4) results into human juvenile polyposis syndrome (JPS) (Haramis *et al*, 2004). JPS is a hereditary disorder characterized by the formation of multiple noncancerous polyps. Although primarily benign, patients with JPS have an increased risk of developing malignant tumors in the digestive tract (Howe *et al*, 1998). Recently, Jaeger and colleagues reported a role for epithelial BMP‐antagonist GREM1 overexpression in hereditary mixed polyposis syndrome (HMPS) (Jaeger *et al*, 2012). However, as most of the epithelial monolayer will be shed into the lumen within a few days, it has been hypothesized that alterations resulting in cancer should affect the stem cell fraction permanently residing at the crypt base. Nevertheless, it has been reported that disruption of the morphogen balance can influence cell fate decisions and make more differentiated cells susceptible for malignant transformation. In the case of HMPS, *GREM1* overexpression leads to formation of ectopic crypt foci (ECF) that grow orthogonally to the crypt/villus axis, thereby mimicking a stem cell niche (Davis *et al*, 2015). Although the importance of the gradient balance has become more evident throughout the years, it remains largely elusive how specific cell types regulate this balance at a molecular level, especially taking into account the variety of cells residing in the microenvironment that can influence the crypt and its gradients (Medema & Vermeulen, 2011). In this issue of *The EMBO Journal*, Horiguchi *et al* (2017) describe a novel mechanism by which ISEMFs control the Wnt/BMP gradient and the ISC niche via ANGPTL2 secretion.

ANGPTL2 is a secreted glycoprotein of the angiopoietin family and is associated with angiogenesis and tissue repair (Kadomatsu *et al*, 2014). Aberrant signaling of ANGPTL2 has been reported to aid chronic inflammatory diseases such as atherosclerosis, metabolic diseases, and diabetes (Horio *et al*, 2014; Oike *et al*, 2005). As the intestine is often afflicted by inflammatory diseases, such as ulcerative colitis and Crohn's disease, Horiguchi *et al* (2017) aimed to study the potential role of ANGPTL2 within the gut. They implemented an *Angptl2* ^−/−^ KO mouse model to study the role of ANGPTL2 in the small intestine and found that ANGPTL2 is dispensable for normal homeostasis. Although the proliferation rates of wild‐type (WT) and *Angptl2*‐deficient mice remained comparable, they did observe a significant reduction in the expression of stem cell markers *Lgr5* and *Ascl2* and in the protein levels of active β‐catenin.

Next, they investigated the role of ANGPTL2 in intestinal regeneration by implementing both the dextran sulfate sodium (DSS) colitis model and radiation. In both models, the expression of *Anpgtl2* was upregulated in WT mice following the injury. In the *Angptl2* ^−/−^ mice, the size of the small intestine and the colon was shortened, no regenerative responses were observed, and a reduced number of viable crypts were detected, suggesting a role for ANGPTL2 in aiding a regeneration response after damage. To identify the cell type producing ANGPTL2, the authors performed immunohistochemical stainings for ANGPTL2, α‐SMA, CD31, and E‐cadherin and observed localization to the mesenchyme and more specifically ISEMFs, not to the epithelium or endothelium. In addition, bone marrow transplantations from *Angptl2* WT mice to KO mice ruled out any involvement of inflammation‐attracted macrophages as the producers of ANGPTL2.

**Figure 1:**
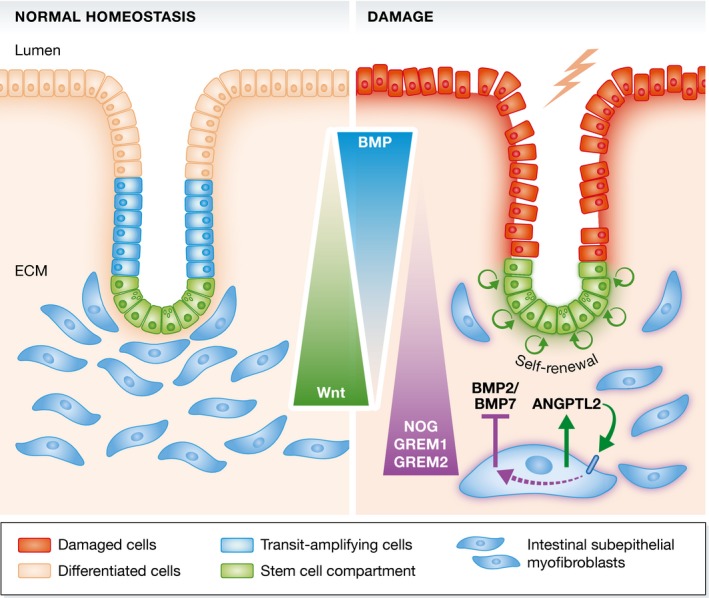
Role of ANGPTL2 in homeostasis and damage repair ANGPTL2 produced by intestinal subepithelial myofibroblasts is expendable for normal homeostasis. However, after intestinal damage, autocrine regulation of ANGPTL2 can shift the crypt/villus gradient toward a more stem cell‐like environment, thereby promoting cell renewal and crypt restoration.

Hence, Horiguchi *et al* (2017) identified ISEMFs as the producers of ANGPTL2 in response to intestinal damage and subsequently aimed to assess the molecular mechanism that eventually lead to crypt regeneration. They discovered that ANGPTL2 secretion induces an autocrine positive feedback loop via integrin α5β1 and the NF‐κB pathway, ultimately leading to upregulation of *BMP2* and *BMP7*. Because epithelial intestinal cells also express integrin α5β1, it was hypothesized that ANGPTL2 could perhaps directly influence β‐catenin levels. However, treatment of WT organoids with recombinant ANGPTL2 did not have any effect on organoid growth, size, or the expression of Wnt pathway genes, suggesting that ANGPTL2 has no direct effect on regeneration. On the contrary, co‐culture, trans‐well, and conditioned medium transfer experiments together with ISEMFs did show a significant effect on organoid growth and size, thereby proposing a solely autocrine role for ANGPTL2.

Altogether, Horiguchi and colleagues elegantly illustrate the role of ISEMFs in regulating the stem cell niche and facilitating crypt regeneration after intestinal damage. They thereby, once again, confirm the vulnerability of the morphogen gradient along the crypt/villus axis. ANGPTL2 produced by the ISEMFs can therefore be seen as an ISC function‐promoting factor that guards the balance of the stem cell niche in times of requirement. Because inflammation can predispose to carcinogenesis, it will be interesting to study the role of ANGPTL2 as a tumor promoter in colon cancer. In line with this hypothesis, ANGPTL2 has already been associated with increased chemo‐resistance and metastasis in some cancers (Endo *et al*, 2012; Horiguchi *et al*, 2014).
